# Trends in Depression and Antidepressant Prescribing in Children and Adolescents: A Cohort Study in The Health Improvement Network (THIN)

**DOI:** 10.1371/journal.pone.0033181

**Published:** 2012-03-13

**Authors:** Linda P. M. M. Wijlaars, Irwin Nazareth, Irene Petersen

**Affiliations:** Department of Primary Care and Population Health, University College London, London, United Kingdom; Chiba University Center for Forensic Mental Health, Japan

## Abstract

**Background:**

In 2003, the Committee on Safety of Medicines (CSM) advised against treatment with selective serotonin reuptake inhibitors (SSRIs) other than fluoxetine in children, due to a possible increased risk of suicidal behaviour. This study examined the effects of this safety warning on general practitioners' depression diagnosing and prescription behaviour in children.

**Methods and Findings:**

We identified a cohort of 1,502,753 children (<18 y; registered with GP for >6 m) in The Health Improvement Network (THIN) UK primary care database. Trends in incidence of depression diagnoses, symptoms and antidepressant prescribing were examined 1995–2009, accounting for deprivation, age and gender. We used segmented regression analysis to assess changes in prescription rates. Overall, 45,723 (3%) children had ≥1 depression-related entry in their clinical records. SSRIs were prescribed to 16,925 (1%) of children. SSRI prescription rates decreased from 3.2 (95%CI:3.0,3.3) per 1,000 person-years at risk (PYAR) in 2002 to 1.7 (95%CI:1.7,1.8) per 1,000 PYAR in 2005, but have since risen to 2.7 (95%CI:2.6,2.8) per 1,000 PYAR in 2009. Prescription rates for CSM-contraindicated SSRIs citalopram, sertraline and especially paroxetine dropped dramatically after 2002, while rates for fluoxetine and amitriptyline remained stable. After 2005 rates for all antidepressants, except paroxetine and imipramine, started to rise again. Rates for depression diagnoses dropped from 3.0 (95%CI:2.8,3.1) per 1,000 PYAR in 2002 to 2.0 (95%CI:1.9,2.1) per 1,000 PYAR in 2005 and have been stable since. Recording of symptoms saw a steady increase from 1.0 (95%CI:0.8,1.2) per 1,000 PYAR in 1995 to 4.7 (95%CI:4.5,4.8) per 1,000 PYAR in 2009.

**Conclusions:**

The rates of depression diagnoses and SSRI prescriptions showed a significant drop around the time of the CSM advice, which was not present in the recording of symptoms. This could indicate caution on the part of GPs in making depression diagnoses and prescribing antidepressants following the CSM advice.

## Introduction

Antidepressants (ADs) are commonly prescribed to children and adolescents for depression, anxiety, and a variety of other disorder [1]. Selective serotonin reuptake inhibitors (SSRIs), first introduced in the late 1980s, were prescribed to children for depression on the basis of effectiveness data from trials on adult psychiatric disorders coupled with other trial data demonstrating the ineffectiveness of tricyclic antidepressants (TCAs) [2]–[5]. In the early 2000s, SSRIs became the preferred treatment for depression in children rather than tricyclic antidepressants (TCAs) [6].

However, doubts have been cast on the use of specific SSRIs in children. In October 2002, the BBC aired an episode of the investigative journalism show ‘Panorama’ which casted doubt on the safety of the SSRI paroxetine. In response to this, the UK Medicines and Healthcare Products Regulatory Agency (MHRA) reanalysed published and unpublished data on paroxetine, and found that the drug failed to demonstrate significant beneficial effects, and was associated with a small increase in suicidal behaviour and ideation [7]. In June 2003, the MHRA hence advised that paroxetine should not be used to treat depression in children younger than 18 years [8]. Following this investigation, the Committee on Safety of Medicines (CSM) reviewed the safety of all antidepressants in children and adolescents and in December 2003 advised against the initiation of all SSRIs, except fluoxetine in children [9]. Fluoxetine is the only drug currently licensed to treat depression in children in the UK as its benefits were deemed greater than its risks [10], [11]. The CSM, however, does recommend psychotherapies, such as cognitive-behavioural therapy, as first-line treatment for children and adolescents with depression. The American Food and Drug Authority (FDA) followed suit in October 2004 and issued a black box warning for all antidepressants prescribed to children [12].

Following the CSM advice, fewer children and adolescents in the UK were prescribed antidepressants in primary care [13]. There was a 48% reduction in the initiation of CSM-contraindicated antidepressants in children between 2002 and 2004. However, the use of fluoxetine and non-SSRI antidepressants in children had not significantly risen during the same period. A similar pattern was found in a study in Australia, where antidepressant use, and SSRI use in particular, in children and adolescents decreased between 2002 and 2005 [14]. In stark contrast, the use of SSRIs and all antidepressants increased significantly in adults during the same period.

Time trends in antidepressant prescribing in children have been described for periods leading up to the CSM advice [1], [13], but this is the first comprehensive study which covers trends in the recording of depression diagnoses and symptoms, and the prescription of antidepressants in children and adolescents from 1995 to 2009 in a large UK general practice database.

## Methods

### Ethics statement

The scheme for THIN to obtain and provide anonymous patient data to researchers was approved by the National Health Service South-East Multicenter Research Ethics Committee (MREC) in 2002 and scientific approval for this study was obtained from CMD Medical Research's Scientific Review Committee in March 2011.

### Data Source

Approximately 98% of the population in the UK is registered with a general practitioner [15]. The Health Improvement Network (THIN) database is one of the largest national collections of primary care data and is broadly representative of the general practice (GP) population in terms of demographics and consultation behaviour [16]. Participating general practitioners from 497 practices enter clinical information on patients, including demographics data, diagnoses, and prescriptions so as to offer a longitudinal medical record for each patient which is available to researchers as anonymised data [17]. Clinical diagnoses recorded by GPs on THIN have been shown to be accurate compared with other reliable sources [18]. The database provides the Townsend score measure of deprivation, a composite measure of social deprivation in quintiles (owner occupation, overcrowding, car ownership, and unemployment) [19]. It is based on patient postal code and linked to UK census data from 2001 for approximately 150 households in that postal area. We analysed data from 1995 to 2009.

### Study population

We identified a cohort of children aged up to 18 years who were registered with a General Practice which was a part of THIN for at least six months between January 1995 and December 2009. Children entered the cohort when they registered with a General Practice, or, the date when their practice joined the THIN scheme and met standards for acceptable levels of data recording [20]. Children remained in the cohort until aged 18 years, transfer out of the practice, date of death or date of last data collection from the practice.

### Measurements

#### Outcome

We examined entries made of diagnoses and symptoms of depression. Depression diagnosis codes ranged from ‘dysthymia’ and ‘mild depression’ to ‘recurrent severe major depression’, but excluded codes that indicated other mental disorder such as psychosis or anxiety. Depression symptoms relate to codes indicating depression but are not certain enough to be classified as a diagnosis, such as ‘symptoms of depression’ or ‘C\O feeling depressed’. We also examined antidepressants BNF codes prescribed by the general practitioner at any dose, except for high dose TCAs (50 mg) that were indicated for nocturnal enuresis [6]. These code lists have been created and used in previous studies and were developed in line with published methods and reviewed by a general practitioner [21], [22].

#### Potential confounders

We included information on age, gender and social deprivation score in our analysis as these are known to be associated with childhood depression and the distribution of these variables may change over the 15 year study period.

#### Statistical analysis

We described the baseline socio-demographic characteristics of the cohort using frequency tables. We calculated annual incidence rates and 95% confidence intervals (CI) for depression diagnoses, symptoms and antidepressant prescriptions by dividing the annual number of incident cases by the total person-years at risk (PYAR) for each year.

Incidence rate ratios adjusted for gender, age and quintiles of Townsend deprivation score) were estimated using a Poisson regression model. The analyses were adjusted for clustering at practice level.

A Lewis plot [23] was used to explore the association between time since registration and incidence rates as prior diagnoses might be registered at or near the time of registration and these ought not to be included in the incidence rates. The Lewis plots revealed that there was an increased rate of depression diagnoses, symptoms and antidepressant prescribing in the first month after registration, after which the rate of recording dropped to a steady state (results not shown). To correct for this, we started follow = up one month after registration.

In order to assess the effects of the CSM advice on antidepressant prescribing, a segmented regression analysis [24] was performed using the Jointpoint regression program (version 3.5.1) from the Surveillance Research Program of the US National Cancer Institute [25]. Jointpoint is statistical software for the analysis of trends using Jointpoint models [26]. This analysis allows for identifying points where there is a change in the linear slope of the trend. The analysis started with the minimum number of jointpoints (i.e., 0 jointpoints, which is a straight line), and tested whether one or more jointpoints (up to 4) were statistically significant and should be added to the model. The models incorporated estimated variation for each point by using the standard error of the rate estimate. After identifying the existence of a change in the trend, a segmented regression was fitted and the result of the best model was shown graphically. Finally, the estimated annual percentage of change (APC) and its corresponding 95% CI was computed for each of those trends by fitting a regression line to the natural logarithm of the rates, using calendar year as a regression variable [27].

All other analyses were conducted in of Stata, version 11.2 (Stata Corp, College Station, Texas).

## Results

**Figure 1:**
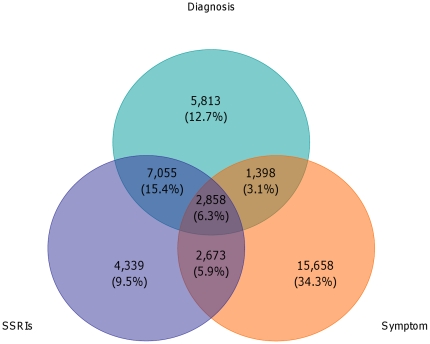
Venn diagram which shows the intersection between depression diagnoses, symptoms and antidepressant prescriptions.

In total, 1,502,753 children up to the age of 18 were registered with their GP for at least one year in The Health Improvement Network (THIN) UK primary care database. Of these children, 45,723 (3%) children had at least one entry of a depressive symptom, diagnosis or antidepressant prescription. Of these children, 17,124 (38%) had a diagnosis of depression, 22,587 (49%) had a record of depressive symptoms, and 25,473 (56%) were prescribed antidepressants, 16,925 of which were SSRIs (Figure 1). Most of these antidepressant prescriptions were for SSRIs: 16,925 (66%), with TCAs representing 7,777 (31%) and other antidepressants 771 (3%) of prescriptions (Table 1). Of the children receiving SSRIs, 4,339 (26%) were not diagnosed with depression or depression symptoms. Similarly, 7,211 (42%) of children diagnosed with depression were not prescribed antidepressants.

**Table 1:** Study population characteristics by antidepressant (AD).

Characteristic of first-time new users	Individuals by AD drug group				
	SSRI(n = 16925)	TCA(n = 7777)	Other (n = 769)	MAOI (n = 2)	Any AD(n = 25473)
Socio-demographic					
Girls	12142 (71.7)	4680 (60.2)	488 (63.5)	1 (50.0)	17311 (68.0)
Most commonlyprescribed drug (# prescriptions)	Fluoxetine (8157)	Amitriptyline (4402)	Mirtazapine (320)	Moclobemide (2)	-
Deprivation quintile					
1 (most affluent)	3252 (19.2)	1631 (21.0)	144 (18.7)	1 (50.0)	5028 (19.7)
2	3063 (18.1)	1451 (18.7)	105 (13.7)	0	4619 (18.1)
3	3456 (20.4)	1646 (21.2)	139 (18.1)	0	5241 (20.6)
4	3732(22.1)	1706 (21.9)	182 (23.7)	0	5620 (22.1)
5 (most deprived)	3195(18.9)	1259 (16.2)	191 (24.8)	1 (50.0)	4646 (18.2)
Not recorded	227 (1.3)	84 (1.1)	8 (1.0)	0	319 (1.3)
Age groups					
3–10 years	179 (1.1)	1577 (20.3)	7 (0.9)	0 (0)	1764 (6.9)
11–14 years	1567 (9.3)	1558 (20.0)	53 (6.9)	1 (50.0)	3192 (19.4)
15–18 years	15179 (89.7)	4642 (59.7)	709 (92.2)	1 (50.0)	20652 (80.7)

Values are numbers (column percentages) unless otherwise indicated.

SSRI = selective serotonin reuptake inhibitor; TCA = tricyclic antidepressant; MAOI = mono-amine oxidase inhibitor; other ADs are: mirtazapine, venlafaxine, flupentixol, duloxetine, nefazodone and reboxetine.

**Figure 2:**
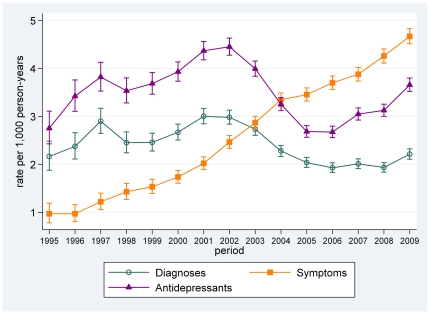
Trends in the incidence of childhood depression, symptoms and antidepressants from 1995 to 2009.

Rates for entries of diagnoses of depression increased from 2.2 (95% CI 1.9–2.5) per 1,000 PYAR in 1995 to 3.0 (95%CI:2.8,3.1) per 1,000 PYAR in 2002, then dropped to 2.0 (95%CI:1.9,2.1) per 1,000 PYAR in 2005 and have since been relatively constant at around 2.0 per 1,000 PYAR (Figure 2). Rates for antidepressant prescribing show a similar pattern: they have gone up from 2.8 (95%CI:2.4,3.1) per 1,000 PYAR in 1995 to 4.5 (95%CI:4.3,4.6) per 1,000 PYAR in 2002, then dropped to rates similar to the initial 1995 rates, but have been increasing again since 2005. Recording of symptoms has seen a dramatic rise from 1.0 (95%CI:0.8,1.2) in 1995 to 4.7 (95%CI:4.5,4.8) per 1,000 PYAR in 2009.

**Figure 3:**
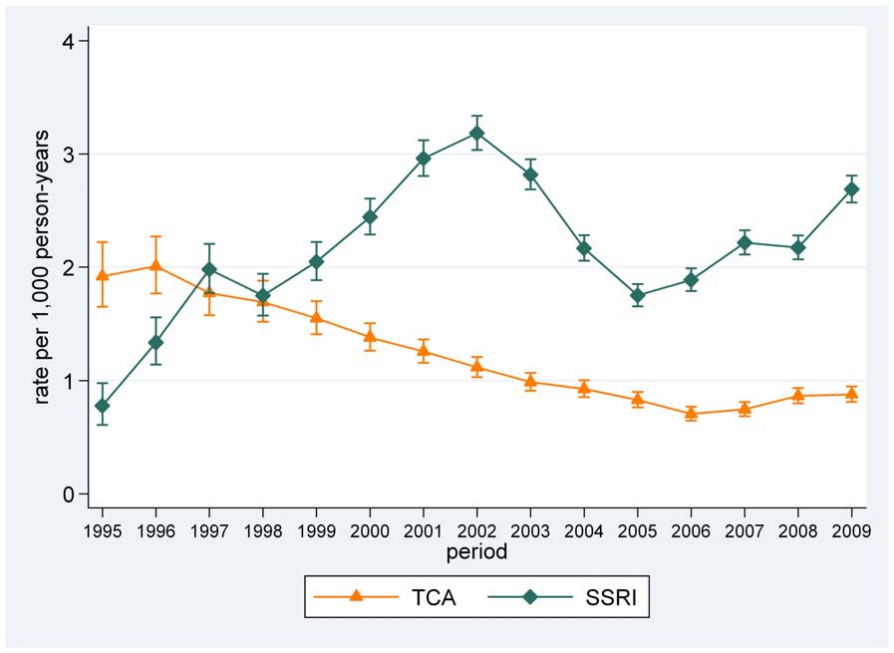
Rates of prescription of Tricyclic Antidepressants (TCA) and Selective Serotonin Reuptake Inhibitors (SSRI) in children.

TCAs were the most common antidepressant prescribed to children in 1995, but by 1999 SSRIs had overtaken them and have been the preferred drug type ever since (Figure 3). However, since 2003 there has been a sharp decline in SSRI prescriptions, with rates decreasing from 3.2 (95%CI:3.0,3.3) per 1,000 PYAR in 2002 to 1.7 (95%CI:1.7,1.8) per 1,000 PYAR in 2005. Since then, rates have gradually started increasing again. TCA prescription rates have gradually decreased since 1995, but stopped decreasing in 2006. Rates for MAOIs and other antidepressants were negligible (results not shown).

In children aged 3–11 years, girls were less likely than boys to be diagnosed as depressed (IRR = 0.79, 95%CI:0.67,0.92), have depression symptoms recorded (IRR = 0.90, 95%CI:0.84,0.95) or be prescribed antidepressants (IRR = 0.63, 95%CI:0.58,0.69; Table 2). In children aged 12–18, girls were more likely than boys to have been diagnosed as depressed (IRR = 2.87, 95%CI:2.77,2.97), have symptoms recorded (IRR = 2.31, 95%CI:2.23,2.39) or have been prescribed antidepressants (IRR = 2.71, 95%CI:2.63,2.80). When comparing age groups, the incidence of all three outcomes in the younger age group (3–11 years old) is only a fraction of that in the older age group (12–18 years old).

**Table 2:** Incidence rate ratios (IRR) for diagnosis and symptoms of depression and antidepressant prescriptions stratified by gender, age group and deprivation.

	Multivariable^a^: stratified by age group: 3–11		Multivariable^a^: stratified by age group: 12–18		Multivariable^a^: stratified by deprivation:Townsend 1 & 2^c^		Multivariable^a^: stratified by deprivation:Townsend 4 & 5^c^	
	IRR (95% CI)	*P* b	IRR (95% CI)	*P* b	IRR (95% CI)	*P* b	IRR (95% CI)	*P* b
Diagnosed depression								
Gender								
Boy	Reference		Reference		Reference		Reference	
Girl	0.79 (0.67–0.92)	0.003	2.87 (2.77–2.97)	<0.001	2.58 (2.44–2.73)	<0.001	2.93 (2.78–3.09)	<0.001
Symptoms of depression								
Gender								
Boy	Reference		Reference		Reference		Reference	
Girl	0.90 (0.84–0.95)	0.001	2.31 (2.23–2.39)	<0.001	1.75 (1.67–1.83)	<0.001	2.12 (2.02–2.22)	<0.001
Antidepressant prescription								
Gender								
Boy	Reference		Reference		Reference		Reference	
Girl	0.63 (0.58–0.69)	<0.001	2.71 (2.63–2.80)	<0.001	2.26 (2.16–2.36)	<0.001	2.57 (2.47–2.69)	<0.001

a Adjusted for calendar year, gender, deprivation, age and for clustering by general practitioner practice using robust standard errors.

b *P* based on Wald test.

c A Townsend score of 1 or 2 represents the most affluent patients, while patients with a Townsend score of 4 or 5 live in the most deprived areas.

Rates for all depression indicators increased with deprivation: children and adolescents in the most deprived quintile were twice as likely to be diagnosed as depressed (IRR = 2.14, 95%CI:2.03.2.26) or be prescribed antidepressants (IRR = 1.91, 95%CI:1.82,2.00) compared to children and adolescent in the least deprived quintile. For depression symptoms, there was an almost 50% increase of recording in the most deprived compared to the most affluent children and adolescents (IRR = 1.43, 95%CI:1.36,1.50).

### Segmented regression analysis

**Figure 4:**
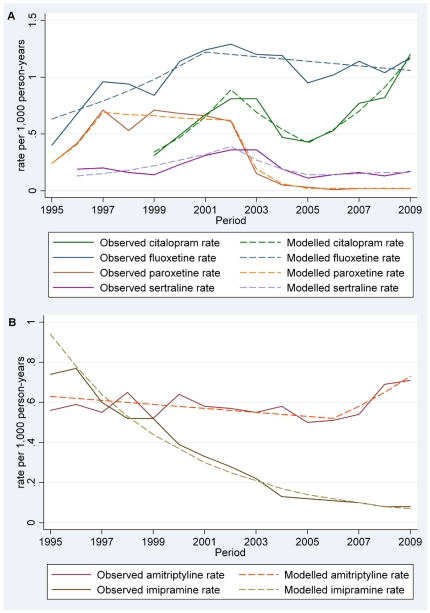
Observed and modelled prescription rates for individual (A) Selective Serotonin Reuptake Inhibitors (SSRIs) and (B) Tricyclic Antidepressants (TCAs).

The Jointpoint analysis suggested for SSRIs as a group, there were two time points were prescription rates changed: 2002 and 2005. Up to 2002 prescription rates for SSRIs had been significantly increasing nearly 16% from 1995–2002 (Table 3, Figures 4A & 4B). However, between 2002 and 2005 the rates were stagnant, followed by a significant increase of nearly 11% from 2005 to 2009 (Table 3).

**Table 3:** Annual percentage change (APC) for selective serotonin reuptake inhibitors (SSRIs) and tricyclic antidepressants (TCAs) as groups and individual drugs.

	*APC 1 (95% CI)*	*Period*	*APC 2 (95% CI)*	*Period*	*APC 3 (95% CI)*	*Period*
SSRIs	15.8* (8.5–23.6)	1995–2002	−19.7 (−36.2–1.0)	2002–2005	10.6* (3.1–18.8)	2005–2009
Fluoxetine	11.6 (−0.4–25.1)	1995–2001	−1.8 (−4.8–1.3)			2001–2009
Citalopram^1^	37.7 (−4.7–98.9)	1999–2002	−22.0 (−51.1–45.4)	2002–2005	29.0* (14.4–45.4)	2005–2009
Paroxetine^2^	67.9 (−27.1–286.9)	1995–1997	−2.2 (−12.2–9.1)	1997–2002	−69.1* (−84.2–39.5)	2002–2005
Sertraline^3^	20.5* (6.1–37.0)	1996–2002	−29.4 (−54.2–9.0)	2002–2005	4.3 (−11.3–22.6)	2005–2009
TCAs	−9.5* (−10.3–8.7)			1995–2006	6.5 (1.0–12.2)	2006–2009
Amitriptyline	−1.8 (−3.8–0.3)			1995–2006	11.9* (2.3–22.3)	2006–2009
Imipramine	−17.1* (−18.6–15.6)					2006–2009

* Annual percentage change (APC) is statistically significant (p<0.05) different from 0.

1 Observations start in 1999 for citalopram as prescription rates were negligible (<10 prescriptions a year) before this year.

2 Observations stop in 2005 for paroxetine as it is only prescribed sporadically (<5 prescriptions a year) after this time point.

3 Observations start in 1996 for sertraline as prescription rates were negligible (<10 prescriptions a year) before this year.

Individual SSRIs followed a similar pattern: fluoxetine, citalopram, paroxetine and sertraline rates all were increasing from 1995 to the early 2000s before showing a temporary decrease, or stall, in prescription rates. Paroxetine was the only SSRI which showed a statistically significant decrease in prescription rates. Rates for citalopram and sertraline started increasing again in 2005, while rates for fluoxetine and paroxetine remained stable (Table 3).

In contrast, rates for TCAs as a group showed a significant decrease between 1995 and 2006, after which there was no significant change. Rates for amitriptyline prescriptions showed a moderate decrease between 1995 and 2006, but started to increase after this point. Imipramine prescription rates showed a steady decline over the entire period.

## Discussion

### Key Findings

To our knowledge, this is the first large paediatric database study to compare depression diagnoses, symptoms and antidepressant prescriptions and the effects the CSM advice over a longer time period in the UK. We have found that prescription rates of SSRIs as a group decreased from 3.2 per 1,000 person-years in 2002 to 1.7 per 1,000 person-years in 2005. More specifically, rates for contra-indicated SSRIs, i.e. citalopram, paroxetine and sertraline, went down during that period, while rates for fluoxetine remained stable and rates for TCAs were not affected. The decline in prescription rates was sharpest for paroxetine. Similar to SSRI prescription rates, rates for depression diagnoses entries decreased from 3.0 per 1,000 person-years in 2002 to 2.0 per 1,000 person years in 2005. Depression symptom recording saw a steady increase over the study period, increasing from 1.0 per 1,000 person-years in 1995 to 4.7 per 1,000 person-years in 2009. Finally, rates for SSRIs as group and citalopram in particular, were increasing after 2005.

The decrease in recording of both depression diagnoses and antidepressant prescriptions after 2002 could indicate caution on the part of GPs in diagnosing depression and prescribing antidepressants following the CSM advice. Moreover, GPs might prefer to record depression symptoms rather than diagnose a child as depressed. The decrease in contra-indicated SSRIs as opposed to fluoxetine strengthens the possibility of a link with the CSM advice.

Although the Jointpoint program points to 2002 as the time point where SSRI rates changed, the observed data shows that rates did not decrease until after 2003, the year when the CSM advice was issued. However, prescription rates could have started decreasing prior to the CSM advice as information regarding the safety and effectiveness of SSRIs was circulating in the specialist community before the advice was issued and could have influenced changes in prescription recommendations. Similarly, the program does not qualify the small dip in fluoxetine rates around 2005 as statistically significant. This dip might be related to the requirement of the US Food and Drug Administration (FDA) to add a black box warning to all antidepressants, including fluoxetine and TCAs, about an increased risk of suicidal behaviour in 2004 [12].

The sharp decline in paroxetine prescription rates could be related to the advice by the MHRA against the prescribing of paroxetine specifically. This advice was issued in June 2003, preceding the overall SSRI advice in December of the same year. It followed a review of randomised controlled trials that showed higher rates of suicidal thought and behaviour (but not completed suicides) in patient who took paroxetine (25 out of 738; 3%), compared to those who took placebo (8 out of 647; 1%; p for difference = 0.01) [8].

The BBC programme that initially started the controversy implied that paroxetine (brand name Seroxat) was addictive, had severe withdrawal symptoms and could increase the risk of suicidal behaviour [28]. This might have led to patients being biased against taking paroxetine as a first line of treatment, and making it difficult to determine whether the sharp decline in paroxetine prescriptions in primary care was due to a negative public opinion of the drug in response to the issues raised in the programme, or the advice issued by the CSM half a year later.

The increase in SSRI prescription rates after 2005 could indicate that concerns about a possible suicidality risk associated with SSRIs have waned. Several studies found no increased risk of suicidality for SSRIs [29], [30], or increases in suicide rates that coincided with decreases in SSRI prescription rates [31]. In the US, 2004 saw the largest single-year increase in suicide rates in adolescents aged 10–19 years [32]. From 1990 to 2003 suicide rates had been decreased by 28%, but in 2004 they had increased with 15% from 6.78 to 7.32 per 100,000 people. This might have led GPs to revaluate prescribing SSRIs to children and adolescents, although doubts continue to exist regarding the safety and effectiveness of SSRIs [33], [34].

### Comparison to other studies

The antidepressant prescription rates we found are similar in size and trend to those found by a General Practice Research Database study which studied prescription rates from 1992 to 2001 [1]. We also found similar age and gender effects. Our results also confirm findings by Murray et al. who found a decrease in SSRI prescribing in between 2003 and 2004, while prescribing rates for fluoxetine remained stable [13]. However, the study by Murray et al. did not assess data for individual drugs, apart from fluoxetine, whereas our study did take different SSRIs into account.

A study based on Australian data also found a decrease in antidepressant use in children, in particular of SSRIs [14]. They also saw a sharp rise of fluoxetine over time, which we did not find in the UK. This might be explained by sertraline being the most commonly prescribed antidepressant in Australia before the SSRI controversy started, whereas fluoxetine was already the drug of first choice in the UK before the CSM advice.

### Main Strengths and Limitations

The main strength of this study is its sample size that enables examination of outcomes separately for girls and boys, and by drug. There is no clear reason to believe the results would differ for the entire population of UK children.

However, there are also limitations. In using data from general practices, few children might have been missed out if their depression was not severe enough to warrant a visit to a GP, or if they were diagnosed outside a general practice setting, e.g. by a child psychiatrist. However, a study on depression in adults found that although incidence rates in the THIN database are lower than depression rates found in epidemiological studies, associations with covariates such as gender and deprivation were similar [35]. Also, non-psychiatric physician's recognition of depression has been found to have a limited sensitivity, but a high specificity [36], [37]. Childhood depression rates might have been underestimated in this study, but trends and associations with other variables are likely to be representative of the general population. Moreover, as we were specifically interested in the effects of the CSM advice in primary care settings, this limitation will have only a minimal effect on our results.

While data on prescriptions is available in the THIN database, there is no information on dispensing and treatment compliance. Thus the antidepressant prescription rates we found might not reflect antidepressant use. However, we aimed to study prescription rates in primary care, so this does not affect our estimates.

### Conclusions

After 2002, general practitioners decreased their prescribing of contra-indicated SSRIs, particularly paroxetine. Rates for fluoxetine, the only SSRI not to be contra-indicated, remained stable. Depression diagnoses mirrored prescription rates and decreased between 2002 and 2005, suggesting caution on the side of GPs. The timing and direction of these trends imply that GPs followed the CSM advice, although it cannot be ruled out that these trends resulted from the negative media attention SSRIs received around the same time. After 2005, rates for all antidepressants, except paroxetine, started recovering. This is in line with results from observational studies that found no increased risk of suicidal behaviour with SSRIs.

## References

[1] Murray ML, de Vries CS, Wong IC (2004). A drug utilisation study of antidepressants in children and adolescents using the General Practice Research Database.. Arch Dis Child.

[2] Stark P, Hardison CD (1985). A review of multicenter controlled studies of fluoxetine vs. imipramine and placebo in outpatients with major depressive disorder.. J Clin Psychiatry.

[3] Hazell P, O'Connell D, Heathcote D, Henry DA (2002). Tricyclic drug for depression in children and adolescents.. Cochrane Database Syst Rev.

[4] Keller MB, Ryan ND, Strober M, Klein RG, Kutcher SP (2001). Efficacy of paroxetine in the treatment of adolescent major depression: a randomized, controlled trial.. J Am Acad Child Adolesc Psychiatry.

[5] Emslie GJ, Heiligenstein JH, Wagner KD, Hoog SL, Ernest DE (2002). Fluoxetine for acute treatment of depression in children and adolescents: a placebo-controlled, randomized clinical trial.. J Am Acad Child Adolesc Psychiatry.

[6] Paediatric Formulary Committee (2010). BNF for Children 2010–2011.

[7] Committee on Safety of Medicines (2003).

[8] Waechter F (2003). Paroxetine must not be given to patients under 18.. BMJ.

[9] Healy D (2003). Lines of evidence on the risks of suicide with selective serotonin reuptake inhibitors.. Psychother Psychosom.

[10] National Institute for Health and Clinical Excellence (2005).

[11] Whittington CJ, Kendall T, Fonagy P, Cottrell D, Cotgrove A (2004). Selective serotonin reuptake inhibitors in childhood depression: systematic review of published versus unpublished data.. Lancet.

[12] FDA website Suicidality in Children and Adolescents Being Treated With Antidepressant Medications.. http://www.fda.gov/Drugs/DrugSafety/PostmarketDrugSafetyInformationforPatientsandProviders/DrugSafetyInformationforHeathcareProfessionals/PublicHealthAdvisories/ucm161679.htm.

[13] Murray ML, Thompson M, Santosh PJ, Wong IC (2005). Effects of the Committee on Safety of Medicines advice on antidepressant prescribing to children and adolescents in the UK.. Drug Saf.

[14] Dean AJ, Hendy A, McGuire T (2007). Antidepressants in children and adolescents - changes in utilisation after safety warnings.. Pharmacoepidemiology and drug safety.

[15] Lis Y, Mann RD (1995). The VAMP Research multi-purpose database in the U.K.. J Clin Epidemiol.

[16] Bourke A, Dattani H, Robinson M (2004). Feasibility study and methodology to create a quality-evaluated database of primary care data.. Inform Prim Care.

[17] Booth N (1994). What are the Read Codes?. Health Libr Rev.

[18] Khan NF, Harrison SE, Rose PW (2010). Validity of diagnostic coding within the General Practice Research Database: a systematic review.. Br J Gen Pract.

[19] Townsend P, Phillimore P, Beattie A (1988). The construction of a measure of deprivation.. Health and Deprivation: Inequality and the North.

[20] Maguire A, Blak BT, Thompson M (2009). The importance of defining periods of complete mortality reporting for research using automated data from primary care.. Pharmacoepidemiol Drug Saf.

[21] Davé S, Petersen I (2009). Creating medical and drug code lists to identify cases in primary care databases.. Pharmacoepidemiology and drug safety.

[22] Davé S, Petersen I, Sherr L, Nazareth I (2010). Incidence of Maternal and Paternal Depression in Primary Care: A Cohort Study Using a Primary Care Database.. Arch Pediatr Adolesc Med.

[23] Lewis JD, Bilker WB, Weinstein RB, Strom BL (2005). The relationship between time since registration and measured incidence rates in the General Practice Research Database.. Pharmacoepidemiol Drug Saf.

[24] Wagner AK, Soumerai SB, Zhang F, Ross-Degnan D (2002). Segmented regression analysis of interrupted time series studies in medication use research.. J Clin Pharm Ther.

[25] National Cancer Institute (2011).

[26] Kim HJ, Fay MP, Feuer EJ, Midthune DN (2000). Permutation tests for joinpoint regression with applications to cancer rates.. Stat Med.

[27] Clegg LX, Hankey BF, Tiwari R, Feuer EJ, Edwards BK (2009). Estimating average annual per cent change in trend analysis.. Stat Med.

[28] Cowen PJ (2002). TV: Panorama: “The Secrets of Seroxat”.. BMJ.

[29] Jick H, Kaye JA, Jick SS (2004). Antidepressants and the risk of suicidal behaviors.. JAMA.

[30] Didham RC, McConnell DW, Blair HJ, Reith DM (2005). Suicide and self-harm following prescription of SSRIs and other antidepressants: confounding by indication.. Br J Clin Pharmacol.

[31] Gibbons RD, Brown CH, Hur K, Marcus SM, Bhaumik DK (2007). Early evidence on the effects of regulators' suicidality warnings on SSRI prescriptions and suicide in children and adolescents.. Am J Psychiatry.

[32] Lubell KM, Kegler SR, Crosby AE, Suicide KarchD (2007). Trends Among Youths and Young Adults Aged 10–24 Years, United States, 1990–2004.. MMWR.

[33] Bridge JA, Iyengar S, Salary CB, Barbe RP, Birmaher B (2007). Clinical response and risk for reported suicidal ideation and suicide attempts in pediatric antidepressant treatment: a meta-analysis of randomized controlled trials.. JAMA.

[34] Hammad TA, Laughren T, Racoosin J (2006). Suicidality in pediatric patients treated with antidepressant drugs.. Arch Gen Psychiatry.

[35] Rait G, Walters K, Griffin M, Buszewicz M, Petersen I (2009). Recent trends in the incidence of recorded depression in primary care.. Br J Psychiatry.

[36] Cepoiu M, McCusker J, Cole MG, Sewitch M, Belzile E (2008). Recognition of depression by non-psychiatric physicians–a systematic literature review and meta-analysis.. J Gen Intern Med.

[37] Kamphuis MH, Stegenga BT, Zuithoff NP, King M, Nazareth I (2011). Recognition of depression in primary care: does it affect outcome?.

